# An Intergenerational Zoom Music Therapy Group During COVID-19

**DOI:** 10.1093/geroni/igab046.2170

**Published:** 2021-12-17

**Authors:** Racheli Lital Gvili

**Affiliations:** Bar-Ilan University, Bar-Ilan, University, Ramat Gan, HaMerkaz, Israel

## Abstract

The COVID-19 pandemic has led to an increase in ageist attitudes and psychological distress and loneliness among older people. The social isolation exacerbated the intergenerational segregation between young and older adults, and has also been expressed within families, since grandparents could not meet their grandchildren in person. The present study involved an intergenerational music intervention, as a vehicle to bridge the gap between grandparents and grandchildren at the COVID-19 pandemic. 41 grandparents aged 56-80, and 45 grandchildren aged 9.9-11.8 took part. Of these, 21 pairs of grandparents and grandchildren participated in a weekly online intergenerational zoom music therapy group for eight weeks, and the rest constituted a waitlist-controlled group. All participants completed the same questionnaires during the same time periods before and after the intervention. The results point to the effectiveness of participation in the sessions in improving intergenerational connections and psychological well-being, and in reducing ageist attitudes and loneliness.

